# Supplementary material for: A model of anaerobic tissue perfusion during trauma—Lactate trajectory curvature can determine recovery
Source: PLoS Comput Biol. 2025 Aug 11;21(8):e1012820. doi: 10.1371/journal.pcbi.1012820 (PMC12360659; doi:10.1371/journal.pcbi.1012820)
Supplement: Attachment — Submitted filename: Response to Reviewers.docx [file pcbi.1012820.s002.docx]

**Reviewers' comments:**

Reviewer's Responses to Questions

**Comments to the Authors:
Please note here if the review is uploaded as an attachment.**

Reviewer #1: Overall the article was well constructed and the research was original. There were minor questions/concerns I had. Is this computational model done in 1D or is it a multi-domain model? I did not see this explicitly defined in the article and it would be helpful for readers to have this stated up front early on in the article. Below are minor edits, mainly concerning the text.

This is a great comment! We found that the introductory paragraph was omitting some detail as well. We have updated the 4^th^ paragraph of the introduction to include these details.

Line 177 - What is meant by "pressure head"? Do you mean cerebral pressure? There is also mention that this blood pressure does not affect the baroreceptor reflex when laying down, so are you taking gravity into consideration with patient-specific heights/vessel lengths and/or angles?

This sentence has been updated to be more clear. By pressure head we meant pressure increase, and the laying down comment was removed because we felt like it was confusing and not included in the scope of the model discussion.

Line 179 - misspelled "use".

Fixed

Line 224 - You say parameter values were chose to fit data well. This is very vague, how did you know the range of parameters to explore when determining these values? From literature? From previous studies? Was there any sensitivity analysis or parameter estimation done to determine these values?

A new parameterization section is included that details parameter values and literature used to estimate them.

Line 242 - Misspelled "etc."

Fixed

Line 247 - "smoothest" seems to be incorrectly used.

Fixed

Line 253 - "begins their recover" seems incomplete. Perhaps authors meant to say "begins to recover" or "begins their recovery".
fixed

Line 283 - "addition" should be "additional" in this context.

fixed

Line 458 - "emergence" should be "emergency" in this context.

fixed

Reviewer #2: The paper by Baird et al. presents a compelling study on anaerobic tissue perfusion during trauma. It introduces and analyzes a mathematical model of hemorrhagic shock and trauma, aiming to construct patient-specific trajectories of serum lactate. The ultimate goal is to support healthcare professionals in making more informed transfusion decisions.

The article addresses a highly relevant topic and presents promising results with significant potential for clinical application. The reviewer believes the paper can be accepted after the following minor revisions are addressed:

- The model includes several nonlinear feedback loops, but some of these are not easy to follow in the current presentation of Section 2. Including a block diagram or more descriptive figures would help clarify the interrelationships between variables across different components of the model.

Great comment, we have included a new diagram showing the model execution over a complete time step, as well as an overview diagram of the software architecture.

- The authors utilize the BioGears engine; however, it is not entirely clear which equations are novel contributions and which are adopted from existing work. The authors should explicitly distinguish between pre-existing elements of BioGears and the modifications or additions made in this study. A clearer discussion of the model’s novel aspects is necessary.

Added a paragraph detailing the new additions to the engine for this study

- The selection of model parameters—whether explicitly stated or implied through numerical values in various equations—lacks sufficient justification. A more thorough discussion of parameter choices, their influence on outcomes, and whether they can be adapted to patient-specific contexts is essential. This would strengthen the assessment of the model’s potential impact in real-world clinical settings.

A parameter section was included in the methods with details on model parameters, how selections were chosen, and a discussion of future work. We have also included addition future work details in the discussion section

Reviewer #3: The authors present a computational model simulating hemorrhagic trauma and recovery, proposing that the curvature of lactate-pH trajectories in phase-space can serve as an indicator of patient recovery. Built on the BioGears platform, the model includes multiple organ system interactions and introduces a logistic curve fit to summarize severity. The study addresses an important clinical modeling gap and introduces some creative computational ideas. However, the validation is largely qualitative, the model modifications are not clearly delineated, the theoretical basis for the curvature approach is underdeveloped, and several methodological details are missing. The claims of clinical applicability are premature given the current evidence, and major revisions are needed to improve the rigor, clarity, and robustness of the work.

Strengths of the Manuscript
• The clinical relevance of modeling hemorrhagic shock and recovery is clear and important.
• The use of curvature of physiological trajectories is a novel computational approach in this setting.
• Leveraging and extending an open-source platform like BioGears promotes transparency and reproducibility.
• The authors are appropriately cautious in acknowledging limitations and outlining future work.
• The logistic curve fitting method provides a potentially useful way to summarize complex simulation results.

Major Criticisms and Questions for Authors
• The validation is qualitative and limited to endpoint lactate levels; can you provide quantitative metrics comparing simulated and experimental time-resolved lactate and pH data?

Excellent observation and we were not able to find human trauma data that was available in time series format for inclusion in this revision. We are a part of an IRB here at UW and can access lactate levels via that IRB for a future study but we weren’t able to complete this data wrangling as part of this submission. We do agree that it is missing in this study and wanted to express our agreement in including it for future work.

• The description of model extensions and modifications to BioGears is vague; can you provide a clear breakdown of what was added or modified compared to the original engine?

A paragraph has been added detailing the original models included for this study

• As far as I can tell, the biological justification for using curvature to define recovery phases is missing; can you explain why maximum curvature points should correspond to transitions in patient state?

Additional text was added to the discussion detailing curvature and patient recovery

• The variability of patient physiology in simulations was artificially narrow; how would your results change with a broader range of patient characteristics such as comorbidities?

A section was included in the results section detailing these observations

• The smoothing and curvature calculation methods may affect the results substantially; have you performed sensitivity analyses to different smoothing window sizes and curvature algorithms?

We have not performed sensitivity analysis for the smoothing window, but do include a discussion of the details in the discussion section

• Details of the simulated patient cohort, including the number of patients per hemorrhage severity and the use of random seeds, are unclear; can you provide more information on cohort design and statistical robustness?

Cohort design was expanded in the results section

• The literature review omits recent computational models of lactate kinetics and trauma physiology; incorporating studies such as Stefanovski et al. (2022, American Journal of Physiology - Endocrinology and Metabolism), Villota-Narvaez et al. (2023, Scientific Reports), and Brooks (2020, The Journal of Physiology) would strengthen the background and show how your work builds upon or differs from existing computational models.

Citations have been added in the introduction with an additional paragraph providing some context
